# Quantification of three-dimensional facial asymmetry for diagnosis and postoperative evaluation of orthognathic surgery

**DOI:** 10.1186/s40902-020-00260-9

**Published:** 2020-05-25

**Authors:** Hua-Lian Cao, Moon-Ho Kang, Jin-Yong Lee, Won-Jong Park, Han-Wool Choung, Pill-Hoon Choung

**Affiliations:** 1 Department of Oral and Maxillofacial Surgery, Dental Research Institute, School of Dentistry, Seoul National University, 101 Daehak-ro, Jongro-gu, Seoul, 03080 South Korea; 2 Onsam Dental Clinic, Seoul, South Korea; 3 Seoul Sarang Dental Clinic, Seoul, South Korea; 4 Department of Oral and Maxillofacial Surgery, Wonkwang University Hospital, Iksan, South Korea; 5 Department of Oral and Maxillofacial Surgery, Chung-Ang University Hospital, Seoul, South Korea

**Keywords:** Facial asymmetry, Three-dimensional computed tomography, Orthognathic surgery

## Abstract

**Background:**

To evaluate the facial asymmetry, three-dimensional computed tomography (3D-CT) has been used widely. This study proposed a method to quantify facial asymmetry based on 3D-CT.

**Methods:**

The normal standard group consisted of twenty-five male subjects who had a balanced face and normal occlusion. Five anatomical landmarks were selected as reference points and ten anatomical landmarks were selected as measurement points to evaluate facial asymmetry. The formula of facial asymmetry index was designed by using the distances between the landmarks. The index value on a specific landmark indicated zero when the landmarks were located on the three-dimensional symmetric position. As the asymmetry of landmarks increased, the value of facial asymmetry index increased. For ten anatomical landmarks, the mean value of facial asymmetry index on each landmark was obtained in the normal standard group. Facial asymmetry index was applied to the patients who had undergone orthognathic surgery. Preoperative facial asymmetry and postoperative improvement were evaluated.

**Results:**

The reference facial asymmetry index on each landmark in the normal standard group was from 1.77 to 3.38. A polygonal chart was drawn to visualize the degree of asymmetry. In three patients who had undergone orthognathic surgery, it was checked that the method of facial asymmetry index showed the preoperative facial asymmetry and the postoperative improvement well.

**Conclusions:**

The current new facial asymmetry index could efficiently quantify the degree of facial asymmetry from 3D-CT. This method could be used as an evaluation standard for facial asymmetry analysis.

## Background

Posteroanterior (PA) cephalometric analysis has been used as a common method to evaluate facial asymmetry. However, there are many inherent limitations to evaluate three-dimensional (3D) skull structures by using two-dimensional (2D) X-ray images. Superimposition of midfacial structures makes it difficult to identify the position of anatomical landmarks [1, 2]. Head position and projection techniques can affect the distortion of images [3]. Therefore, Grummons et al. reported that frontal cephalometric analysis could not be used for either quantitative or comparative analysis of facial asymmetry [4].

In 2D analysis of facial asymmetry, the establishment of an accurate reference line is the most important step because the degree of facial asymmetry is determined by the reference lines. Many researchers proposed various reference lines [2, 5, 6]. However, all the proposed reference lines could not be the gold standard. As the reference lines are established according to the clinician’s preferences, distortion of the degree of facial asymmetry by the reference lines cannot be excluded.

As we have got more precise images from the three-dimensional computed tomography (3D-CT), many professionals have been applied 3D-CT to assess facial asymmetry [7–13].

To assess facial asymmetry based on 3D-CT, various anatomical landmarks, lengths, and angles that were used in 2D analysis have been applied in 3D analysis [11, 14]. Although 2D cephalometric data is accustomed to the orthodontists and oral and maxillofacial surgeons, the measurements which have been used in the cephalometric analysis do not have the same values on the 3D reconstruction model. Some landmarks, such as orbitale and sella, are shown as a line or a point in the space, so these landmarks should be re-defined in 3D skull structures. Therefore, there should be a new method to evaluate 3D facial asymmetry based on 3D environments [15].

Katsumata et al. proposed the facial asymmetry index based on 3D-CT, and this method had the advantage to represent the degree of 3D facial asymmetry as a numerical value [13]. Some other researchers applied and revised this method to evaluate the facial asymmetry on the 3D basis [13, 16–18]. To use this method in the treatment planning and postoperative evaluation of orthognathic surgery, reproducible identification of reference landmarks is important because errors in the identification of reference landmarks are considered as the major sources of errors in cephalometric analysis [19]. These studies did not suggest the method that the positional data of reference landmarks, which were identified on the preoperative CT images, were maintained on the follow-up CT images. Additionally, facial asymmetry index in these studies did not represent the direction of facial asymmetry. Therefore, modifications are required for this method to be used for diagnosis and postoperative evaluation of orthognathic surgery.

In many studies, to evaluate facial asymmetry on the 3D basis, three-dimensional reference planes (horizontal, midsagittal, and coronal reference planes) were established and *x*,*y*,*z* coordinates of the landmarks to the reference planes were used [12, 13, 16, 20–22]. However, the method of establishing reference planes according to the clinician’s preferences could make the same problem which happened in 2D analysis. The use of a coordinate system based on selected reference planes might have greater possibilities of distortion in representing the degree of facial asymmetry, so the clinicians should be careful to determine the reference planes.

This study proposed the method to quantify the degree of three-dimensional facial asymmetry only by using the distances between the anatomical landmarks. In the normal standard group, the reference facial asymmetry index of ten anatomical landmarks was obtained. This method was applied to the patients who had undergone orthognathic surgery. It was checked that the facial asymmetry index showed the preoperative facial asymmetry and postoperative improvement well.

## Materials and methods

### Subjects

A group of 25 male patients (mean age, 21.5 years) who had undergone 3D facial CT was selected as the normal standard group. The selection criteria of the normal standard group were described below. All patients had normal occlusion and showed no facial asymmetry on clinical examination by two oral and maxillofacial surgeons (M-H.K and J-Y L). First molars and central incisors of the subjects were present and in function. The position of upper dental midline was coincided with the facial midline. The midsagittal plane which passed through nasion, sella, and midpoint of both frontozygomatic suture points (MidZ) was established. The subjects which had the length of the perpendicular line from menton to the midsagittal plane under 4 mm were included as the normal standard group [21, 23]. The diagnosis of the patients was temporomandibular disorder and 3D facial CT images were taken to examine the condylar shape and resorption, but the patients who had normal condylar shape and no evidence of resorption were included in this study.

### Selection of reference and measurement landmarks

**Fig. 1:**
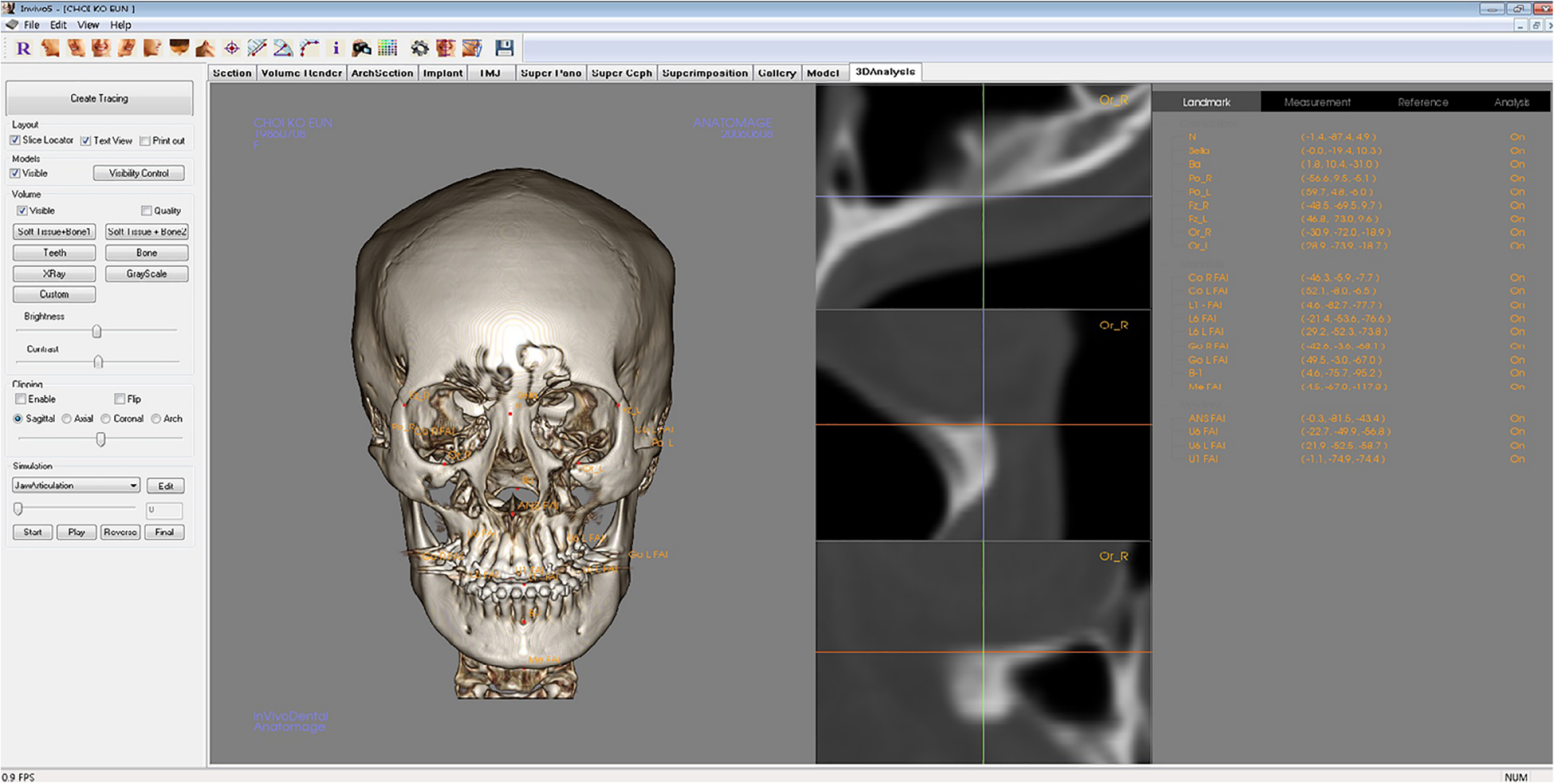
The *x*,*y*,*z* coordinate data of 5 reference landmarks—nasion, sella, midpoint of both *Z* points, frontozygomatic suture point, and porion—and 10 measurement landmarks were obtained by using 3D simulation software

The CT machine in this study was Somatom Sensation 10 (Giemens, München, Germany) and the slice thickness in the reformatted images was 1 mm. Five anatomical landmarks—nasion (N), sella (S), frontozygomatic suture point (*Z* point), midpoint of both *Z* points (MidZ), and porion (Po)—were selected as reference points (Table 1). Measurement points were 10 anatomical landmarks which consisted of 5 midsagittal landmarks—anterior nasal spine (ANS), upper incisor (U1), lower incisior (L1), B point, and menton (Me)—and 5 bilateral landmarks—orbitale (Or), condylion (Co), gonion (Go), upper 1st molar (U6), and lower 1st molar (L6). The *x*,*y*,*z* coordinates were obtained from the 3D simulation software, InVivo5 (Anatomage, Inc., San Jose, USA) (Fig. 1).

**Table 1:** Anatomical landmarks

Landmark		Definition
Landmarks as reference points	S (sella)	Center of the pituitary fossa
	N (nasion)	Nasofrontal suture at the midline
	MidZ (midpoint of *Z*)	Midpoint of the line between both *Z* points
	*Z* point	The most inferior point on the zygomaticomaxillary suture
	Po (porion)	The most superior point of the external auditory meatus
Landmarks as midsagittal measurement points	ANS	Anterior nasal spine
	U1	The superior of the contact point between the upper central incisors
	L1	The superior of the contact point between the lower central incisors
	B point	The deepest point in the bony concavity in the mandibular midline
	Me (menton)	The lowest border of the mandible
Landmarks as bilateral measurement points	Or (orbitale)	The lowest points of the orbital rim
	Co (condylion)	The most superior points of the condyles
	Gonion	The most inferior and posterior points at the angles of the mandible
	U6	The central points of the pulp cavity at the upper first molar
	L6	The central points of the pulp cavity at the lower first molar

### Calculation of facial asymmetry index on the bilateral landmarks

**Fig. 2:**
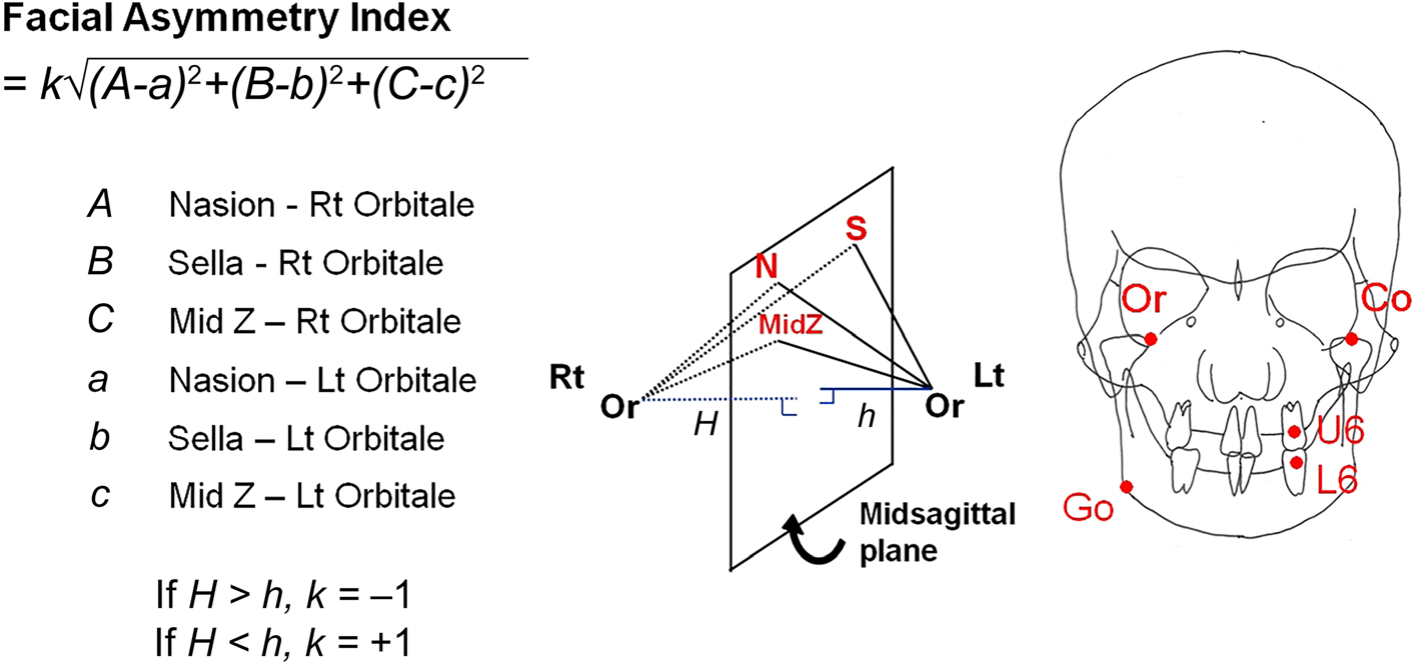
Calculation of facial asymmetry index on orbitale (Or). The formula was designed to show the degree of facial asymmetry by using the difference between the distances from the reference point to bilateral measurement landmarks. If both orbitales were on 3 dimensionally symmetric positions to the 3 reference landmarks (nasion, sella, MidZ), the value of facial asymmetry index was 0. As the asymmetry of both orbitales increased, the facial asymmetry index on orbitale increased. The formula was designed so that the right deviation had a negative value while the left deviation had a positive value. The facial asymmetry indices were calculated on the 5 bilateral landmarks—orbitale (Or), condylion (Co), gonion (Go), upper 1st molar (U6), lower 1st molar (L6)

The distances between the reference points and measurement points were calculated. On the bilateral landmarks, the difference in the distance from each reference landmark (sella, nasion, and MidZ) to bilateral measurement landmarks was calculated and the sum of the square value of each difference was obtained. The root value of the sum was defined as facial asymmetry index (Fig. 2). If both measurement landmarks were on 3 dimensionally symmetric position to the 3 reference landmarks, facial asymmetry index represented 0. As the difference of three-dimensional position of both bilateral landmarks increased, in other words, as the asymmetry increased, the facial asymmetry index increased. The lengths of a perpendicular line from bilateral landmarks to the plane which passed through the 3 reference landmarks (N, S, and MidZ) were compared and the direction of deviation was defined as the side which had longer perpendicular line. The formula was designed so that the right deviation had a negative value while the left deviation had a positive value.

### Calculation of the facial asymmetry index on the midsagittal landmarks

**Fig. 3:**
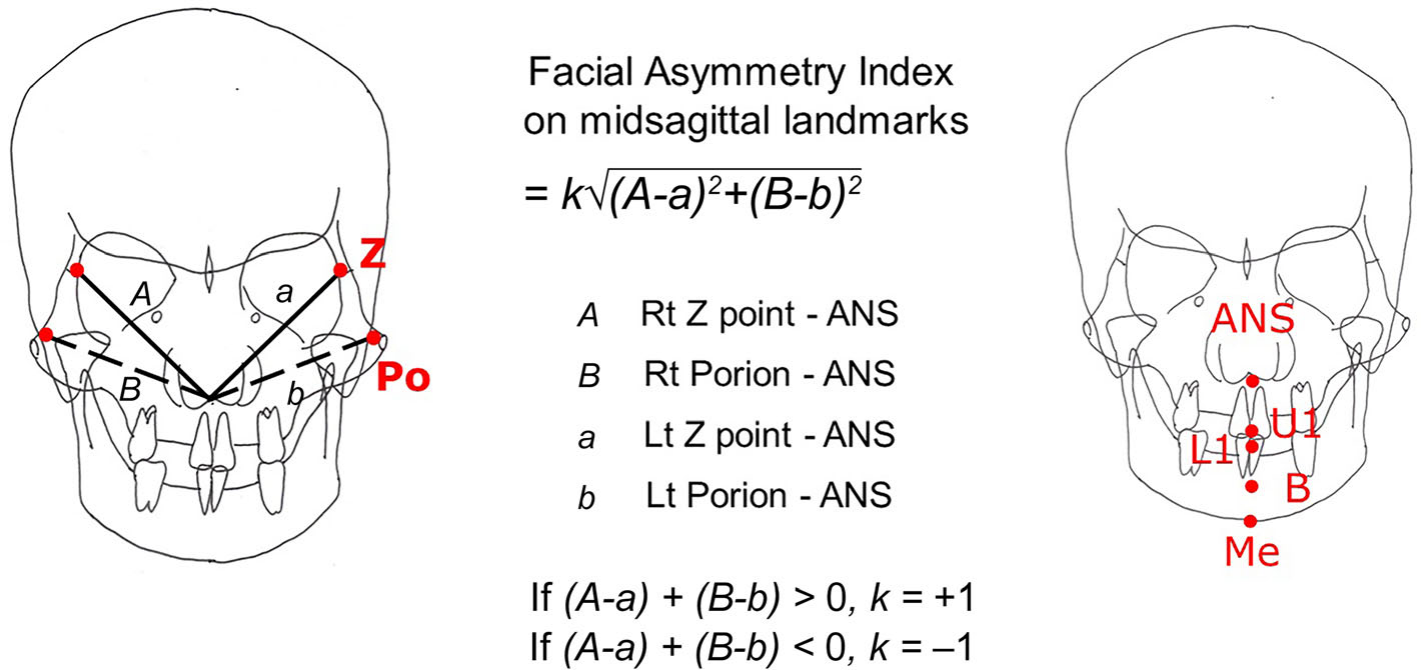
Calculation of facial asymmetry index on ANS. The differences of distances between 2 reference points—frontozygomatic suture point (*Z* point) and porion (Po)—and ANS were measured. The sum of the square value of each difference was obtained, and we defined the root value of the sum was defined as facial asymmetry index on the midsagottal landmarks. If ANS was on the true midsagittal plane, the facial asymmetry index indicated 0

Frontozygomatic suture point (*Z* point) and porion (Po) which were located bilaterally on the cranial bone were selected as reference landmarks to get the facial asymmetry index on the midsagittal landmarks. The difference of the distances between 2 reference points and 5 measurement points was measured. The sum of the square value of each difference was obtained. The root value of the sum was defined as facial asymmetry index on the midsagittal landmarks. The formula was designed so that the right deviation had a negative value while the left deviation had a positive value (Fig. 3).

### Clinical application of facial asymmetry index

**Fig. 4:**
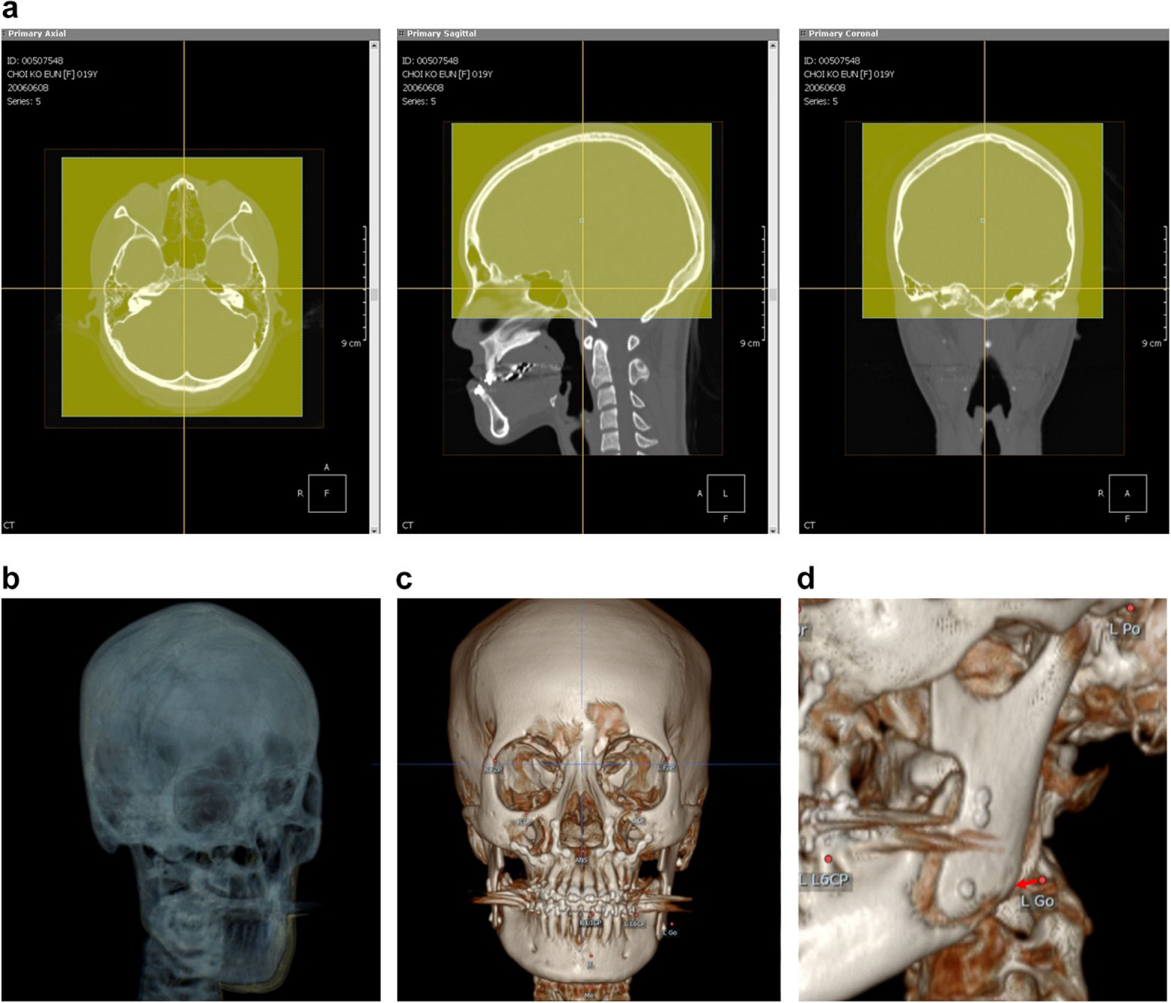
In three-dimensional simulation software, serial 3D-CT data were superimposed automatically on the best fit of cranial base structures by using volume registration (**a**, **b**). Three-dimensional positions of landmarks which were selected on preoperative CT images were saved and loaded on postoperative CT images (**c**). If the positions of measurement landmarks were changed after surgery, it can be moved to the new position on the software and the new coordinate data can be saved without the positional change of reference landmarks (**d**)

For the application of facial asymmetry index to evaluate the postoperative changes of the patients, serial 3D-CT images of the same patient should be taken. For an accurate evaluation, the position of reference point which was selected on the preoperative CT images should be maintained on the postoperative CT images. 3D simulation software, OnDemand 3D (Cybermed, Inc., Seoul, Korea), was used for this purpose. In the VCeph 3D module of this software, serial 3D-CT data were superimposed automatically on the best fit of cranial base structures by using volume registration [20, 24]. Three-dimensional position of landmarks that were selected on preoperative CT images was saved and loaded on postoperative CT images. Therefore, measurement landmarks can be identified on postoperative CT images without positional change of 5 reference landmarks. Therefore, observer-dependent error, which happened between the serial CT images, can be excluded (Fig. 4). In case there were positional changes of landmarks by surgery, the positions of measurement landmarks were moved to the new positions on the software and the coordinate data of the new position were saved (Fig. 4).

In this study, all the personal information was erased from the CT data except gender and age. All the CT data were changed to the anonymized files. After this procedure, these files were used for this study. This study was reviewed and approved by the institutional review board at School of Dentistry, Seoul National University, Seoul, Korea (No.S-D20120009).

### Reliability of facial asymmetry index

To prevent the inter-observer error, all measurements were performed by one author (M-H.K). To evaluate the reproducibility of facial asymmetry index, 10 patients were selected randomly from the control group. One hundred facial asymmetry indices from 10 patients were measured twice during an interval of 2 weeks. A paried *t* test between double measurements was performed with SPSS for Windows (version 17.0, SPSS Inc., Chicago, USA). The method errors were calculated according to the formula SE = √(∑*d*^2^/2*n*) (*d* is the difference between double measurements and *n* is the number of paired double measurements) [25].

## Results

The intra-observer precision error of facial asymmetry index was 0.56. There was no significant difference between the original and repeated measurements in facial asymmetry index (*p* = 0.7567).

### Normal standard group

**Fig. 5:**
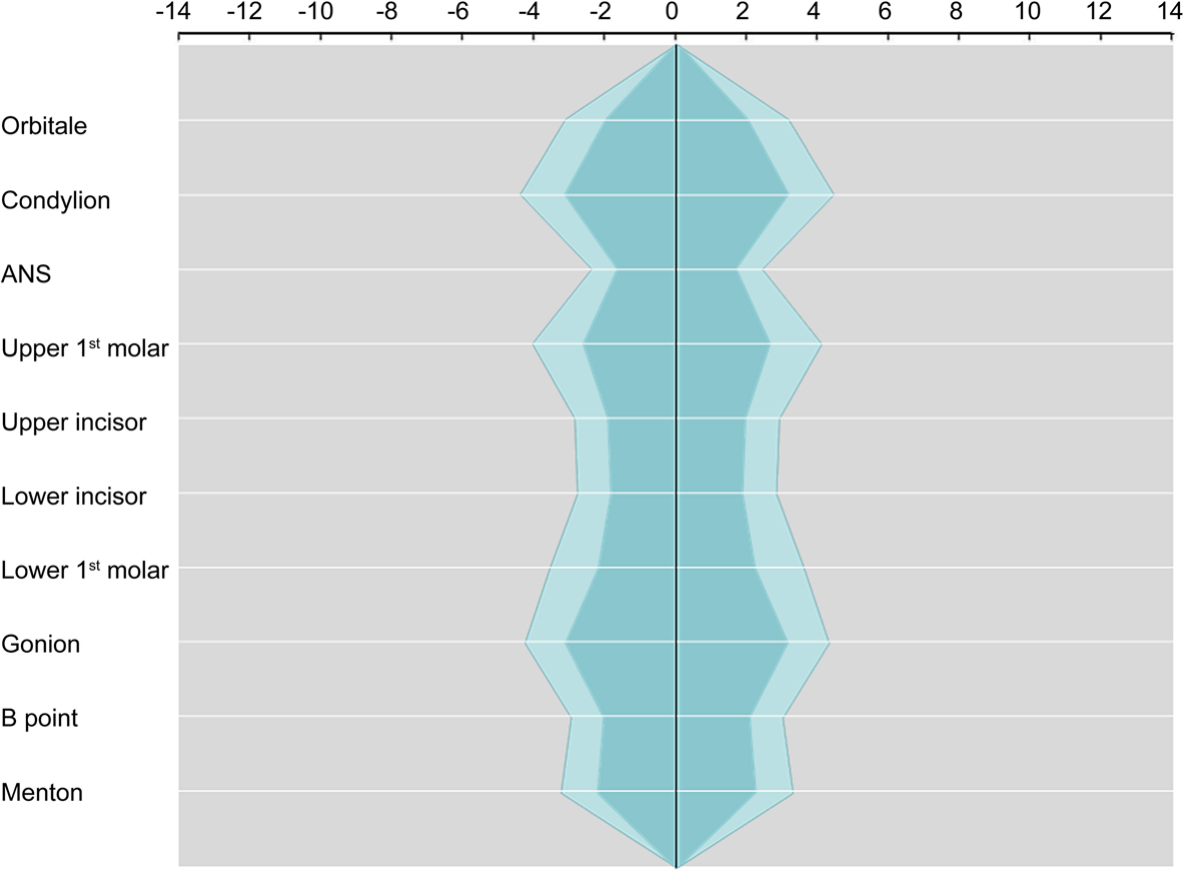
Polygonal chart of the reference facial asymmetry index. The inner green area indicates the mean asymmetry indices and the outer light green area indicates the mean plus the standard deviation value (*p* = 0.05). Right side deviation has a negative value, while left side deviation has a positive value

The facial asymmetry indices were calculated on 10 anatomical landmarks. The absolute value of asymmetry indices ranged from 1.77 to 3.38 (Table 2). The asymmetry index of condylion was the largest, while the asymmetry index of anterior nasal spine (ANS) was the smallest. The polygonal chart of the reference facial asymmetry index was drawn for visualization. The inner green line indicated the mean asymmetry indices and the outer light green line indicated the mean plus the standard deviation value (*p* = 0.05). The green and light green areas were defined as facial symmetry and the outer gray area was defined as facial asymmetry. The right side deviation had negative value, while the left side deviation had positive value (Fig. 5).

**Table 2:** Reference facial asymmetry index in the normal standard group (*n* = 25, absolute value)

Landmark	Mean	SD
Orbitale	2.12	1.28
Condylion	3.38	1.41
Anterior nasal spine	1.77	0.83
Upper first molar	2.81	1.61
Upper incisor	2.05	1.07
Lower incisor	1.97	1.07
Lower first molar	2.35	1.53
Gonion	3.36	1.29
B point	2.18	1.05
Menton	2.37	1.17

### Clinical application of facial asymmetry index

#### Case 1

**Fig. 6:**
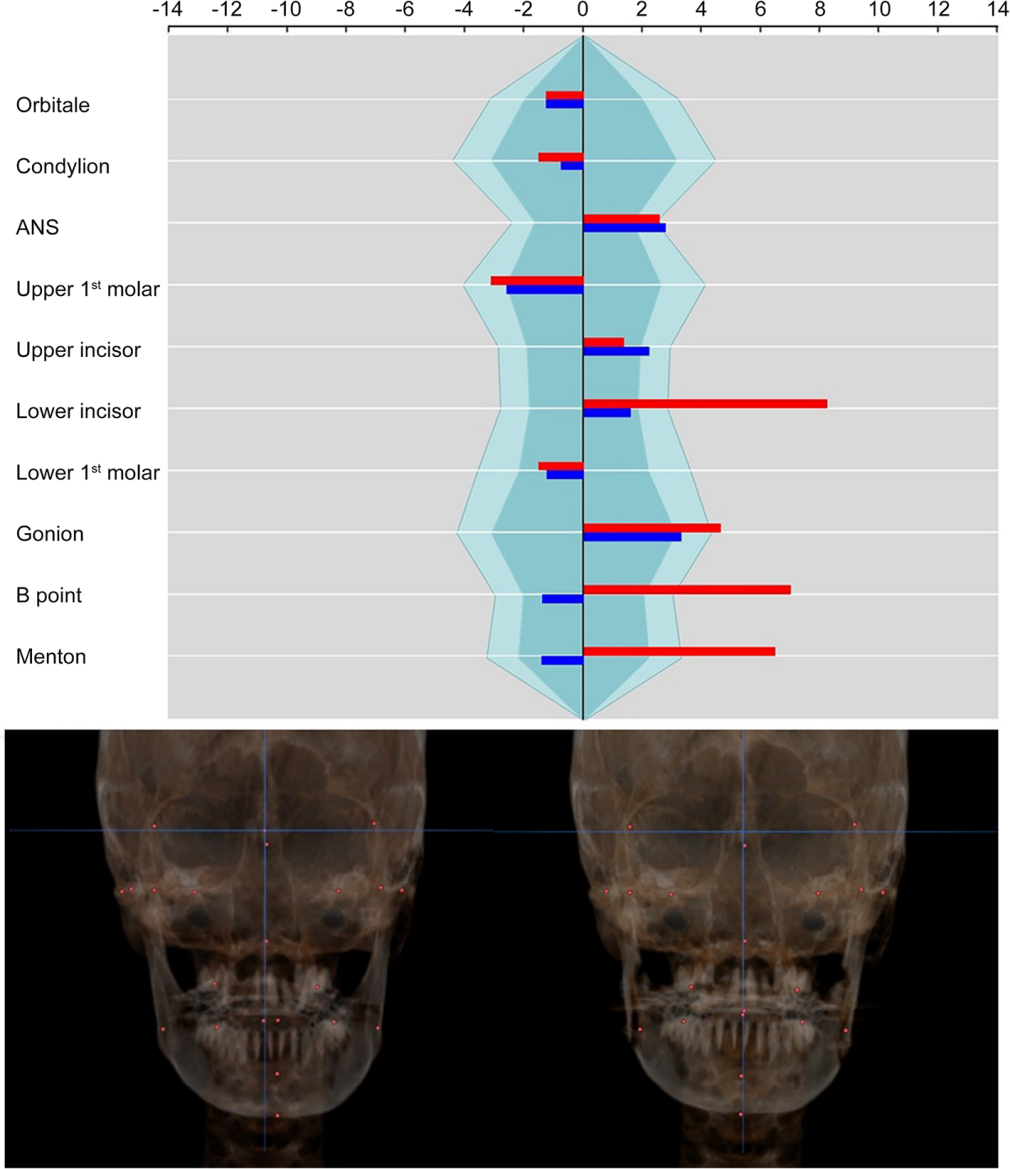
Polygonal chart and 3D reconstruction images of case 1. The asymmetry index on orbitale was not changed because the 3D coordinate data of reference landmarks were maintained on the postoperative images. Asymmetry indices on maxillary landmarks almost did not change because the maxillary surgery did not include the canting correction or midline correction. Asymmetry indices on mandibular landmarks were greatly improved (red: before surgery, blue: after surgery)

The patient was a 20-year-old female with mandibular prognathism and facial asymmetry. Mandible was deviated to the left side by 3 mm and maxillary canting was not found on clinical examination. She underwent orthognathic surgery which consisted of Le FortIosteotomy and intraoral vertico-sagittal split ramus osteotomy (IVSRO). The surgical plan included 3 mm of advancement and 2 mm of superior impaction of the maxilla and asymmetric setback surgery of the mandible (right side, 11 mm; left side, 5 mm) and grinding of chin point. Facial asymmetry index on orbitale was not changed because the surgery did not include the orbital area and the 3D positions of reference landmarks were maintained on the postoperative images. On the maxillary landmarks, the asymmetry indices almost did not change on ANS, U6, and U1. It was because the maxillary surgery did not include midline correction or canting correction. On the other side, the facial asymmetry indices on mandibular landmarks were greatly changed on L1 (from 8.56 to 1.65), B point (from 7.28 to − 1.45), and Me (from 6.74 to − 1.48) (Fig. 6). It showed that mandibular asymmetry was improved by asymmetric setback surgery.

#### Case 2

**Fig. 7:**
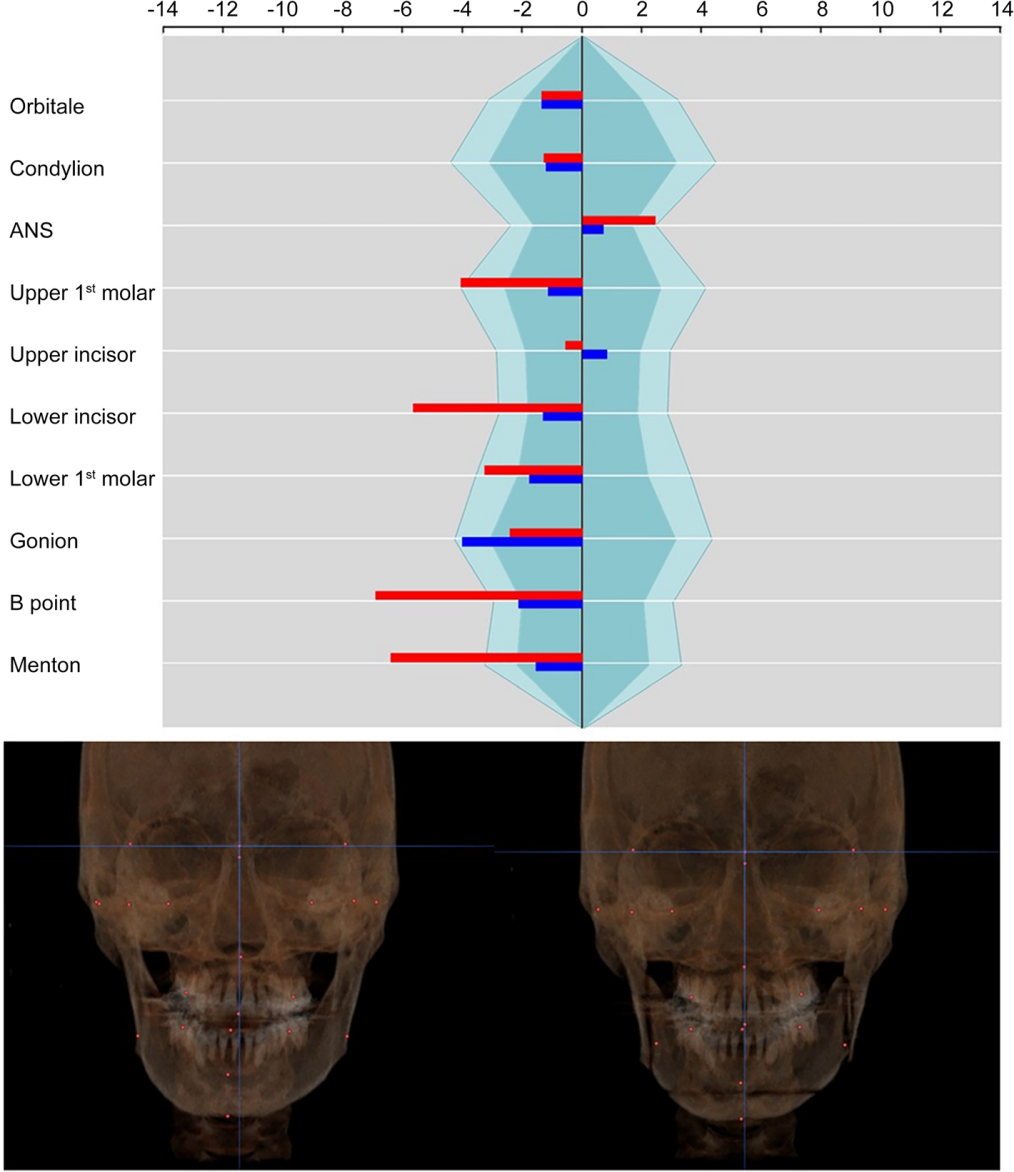
Case 2. The asymmetry index on U6 and U1 was improved by canting correction and midline correction of the maxilla. Mandibular asymmetry was improved by asymmetric setback surgery (red: before surgery, blue: after surgery)

The patient was a 26-year-old male with mandibular prognathism and facial asymmetry. Three millimeters of maxillary canting was present, which the right side was longer than the left side. Maxillary midline was deviated to the right side by 2 mm and the mandible was deviated to the right side by 5 mm. The surgical movement of maxillary surgery consisted that 3 mm of canting correction, 4 mm of posterior impaction, and midline correction to the left side by 1.5 mm. For mandible, asymmetric setback (right side, 12 mm; left side, 18 mm) and advancement genioplasty were done. On the maxillary landmarks, the asymmetry index on U6 was improved from − 3.75 to − 1.05 by canting correction, and the asymmetry index on U1 was changed from − 0.53 to 0.76 by midline correction. The asymmetry indices on mandibular landmarks were improved on L1(from − 5.22 to − 1.22), B point (from − 6.38 to − 1.98), and Me (from − 5.90 to − 1.44) (Fig. 7).

#### Case 3

**Fig. 8:**
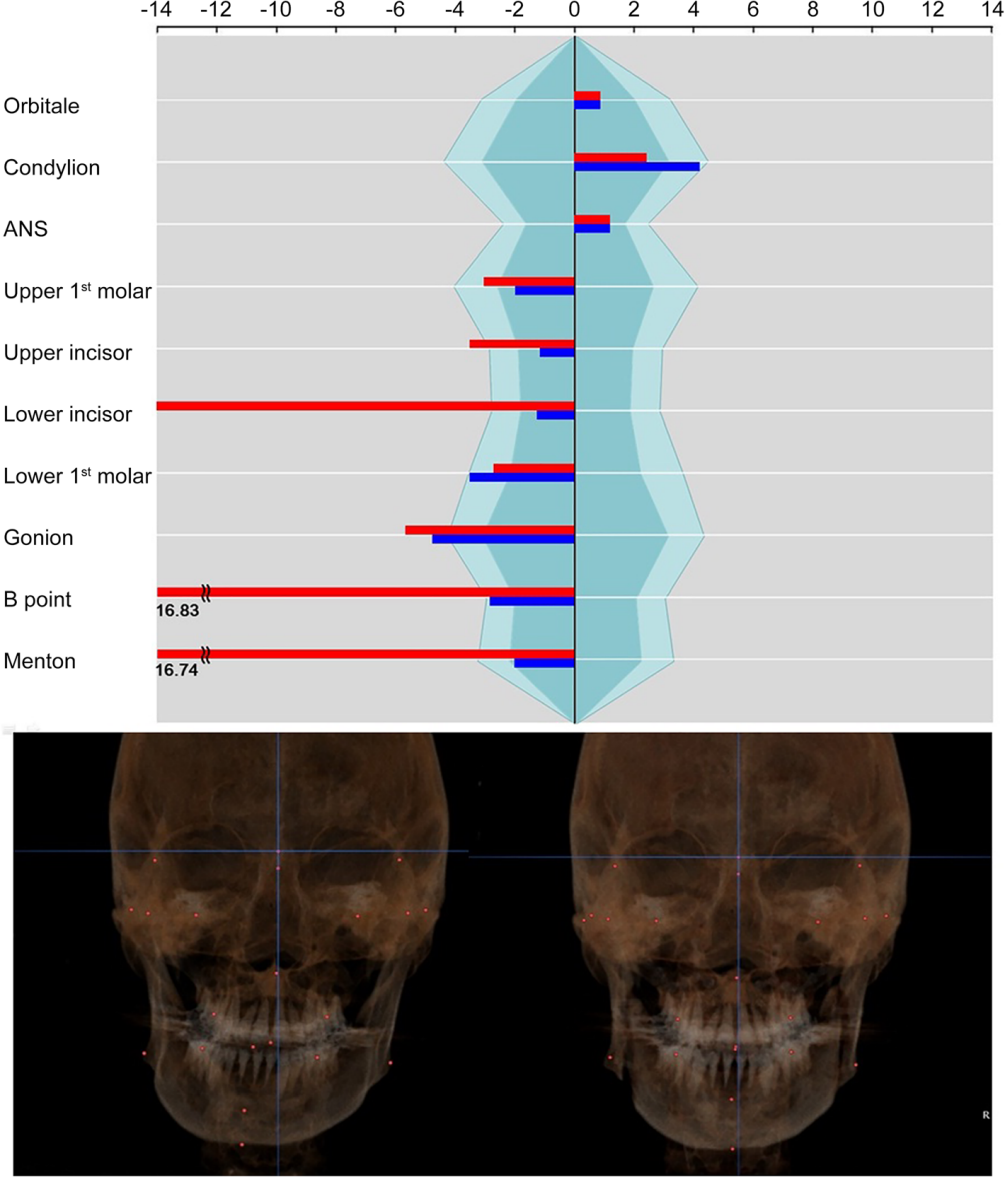
Case 3. The asymmetry index on U1 was improved by midline correction of the maxilla. Severe mandibular asymmetry was improved, but asymmetry on gonion remained (red: before surgery, blue: after surgery)

The patient was a 25-year-old male with severe facial asymmetry and mandibular prognathism. On clinical examination, the maxillary midline was deviated to the right side by 2 mm. The mandible was deviated to the right side by 7 mm. Three-millimeter canting of the maxilla was present, which the left side of the maxilla was longer than the right side. The surgical movement of maxillary surgery consisted 3 mm of canting correction, 2 mm of posterior impaction, and 2 mm of midline correction to the left side. For the mandible, asymmetric correction via IVSRO (right side, advance 1 mm; left side, setback 11.5 mm) was done. On the maxillary landmarks, the asymmetry indices were improved on U6 (from − 3.04 to − 2.00) and U1 (from − 3.52 to − 1.17). The asymmetry indices on mandibular landmarks were greatly improved on L1 (from − 14.00 to − 1.25), B point (from − 16.83 to − 2.83), and Me (from − 16.74 to − 2.03) (Fig. 8).

## Discussion

Basic treatment goal in patients with facial asymmetry is the correction of the deviated midline of the maxilla, mandible, and chin point. On the other hand, Yanez-Vico et al. reported that the angle of the mandibular ramus, on both frontal and lateral planes, determined apparent facial asymmetry [17]. Hwang et al. also commented that some patients complained of mandibular asymmetry even after successful correction of chin deviation, so the operators should pay attention to the improvement of the condylar axis, such as frontal and lateral ramal inclination [11]. Because there are a lot of limitations to evaluate 3D skull morphology by using frontal and lateral cephalometric X-rays, for successful correction of facial asymmetry, 3D evaluation of facial asymmetry by using 3D-CT is necessary. Various methods have been reported to evaluate facial asymmetry based on 3D-CT. Damstra et al. suggested a combined 3D and mirror-image analysis for the diagnosis of facial asymmetry [26]. The others reported the evaluation method using facial asymmetry index [13, 16, 17].

To use these methods, identifying reference landmarks and establishing appropriate reference planes are crucial steps for the evaluation of facial asymmetry. In 3D environments, when the reference planes are established, clinicians should consider not only the horizontal and vertical position of reference planes, but also the rotational position such as yaw, pitch, and roll [27].

Yanez-Vico et al. used mid-dorsal position of the foramen magnum, bilateral points of the external auditory meatus, and foramen spinosums which were located in the middle and posterior cranial base because they thought this area might be the most stable area during development [17].

Katsumata et al. used the plane which passed through sella, nasion, and dent as a midsagittal reference plane [13]. And two more planes perpendicular to this midsagittal plane were selected as horizontal and coronal reference planes.

In this study, nasion, sella, and MidZ were selected as the midsagittal reference points. MidZ was used as a reference point instead of basion or dent. If the landmarks like basion and dent which were located on the posterior part of the cranium were selected as the reference points, posterior cranial bone asymmetry could affect the evaluation of anterior mandibular asymmetry, such as chin point deviation. In PA cephalometric analysis, Trpkova et al. reported that the perpendicular line through midpoints between pairs of orbital landmarks showed excellent validity as the vertical reference line [2]. In CBCT analysis, Park et al. used bilateral *Z* points and orbitale in 3D reconstruction images and reported that the transverse reference line using these landmarks might be used even in patients with a severe asymmetry of the maxilla when this was used with reference to the clinical photos [28]. If the clinicians chose the posterior cranial landmarks as reference landmarks, it is difficult for clinicians to use them on clinical examination because they cannot see and measure the landmarks. If the clinicians used orbital landmarks as reference landmarks, the clinicians are able to compare the degree of asymmetry on CT images with that on clinical examination and photos. If the patient has obvious asymmetry in the orbital area, it is better to allow orbital asymmetry in setting the reference plane rather than using posterior cranial landmarks.

Therefore, the authors used MidZ as a midsagittal reference point instead of posterior cranial bone landmarks like basion or dent.

To define the inclusion criteria for the control group, previous researches about PA cephalometric analysis were used. Some researchers showed that the critical distance of menton that distinguished symmetry from asymmetry was approximately 4 mm [21, 23]. So, in this study, the patients who had the length of the perpendicular line from menton to the Na-S-MidZ plane under 4 mm were included as the control group.

Kwon et al. proposed the similarity index to evaluate three-dimensional asymmetry [29]. They evaluated the symmetry of the mandible using a mirror image. When overlapping the left and right of the mandible, the overlapping part is expressed by similarity index. The closer the similarity index is to zero, the more symmetrical it is. This is also a good way to evaluate facial symmetry. However, this method evaluates symmetry by dividing the mandible into two parts, ramus and body. Therefore, there is a limit in evaluating which anatomical landmarks have asymmetry.

To evaluate the preoperative facial asymmetry and postoperative improvement, reproducible identification of the landmarks is important [30]. In the studies of Katsumata and Yanez-Vico, there is no solution how to maintain the positional data of reference landmarks on the serial CT images. If the reference landmarks should be re-identified on the follow-up CT images, the index value of landmarks, such as orbitale, which were not changed by the treatment could be changed. Therefore, errors in identifying the landmarks and the change following the treatment could be mixed and represented as an index. Therefore, it may adversely affect the accuracy of the evaluation.

In this study, superimposition of serial CT images was done on the best fit of cranial base structures. In the VCeph 3D module of OnDemand 3D software, the positional data of selected landmarks on the preoperative CT images were saved and loaded on the postoperative CT images. Therefore, the position of reference landmarks was maintained on the postoperative 3D model. The measurement landmarks, which were changed following surgery, were moved to the new position on the postoperative 3D model, and the new positions were also checked on the multiplanar reconstruction (MPR) images. This method was able to minimize the error of identifying the landmarks in the follow-up CT images and improve the accuracy of postoperative evaluation.

## Conclusions

In this study, the current new facial asymmetry index was proposed and it could efficiently quantify the degree of facial asymmetry from 3D-CT. This method could be used as an evaluation standard for facial asymmetry analysis.

## Data Availability

The datasets used during the current study are available from the corresponding author on reasonable request.

## Abbreviations

FAI: Facial asymmetry index
3D-CT: Three-dimensional computed tomography

## References

[1] Pirttiniemi P, Miettinen J, Kantomaa T (1996). Combined effects of errors in frontal-view asymmetry diagnosis. Eur J Orthod.

[2] Trpkova B, Prasad NG, Lam EWN, Raboud D, Glover KE, Major PW (2003). Assessment of facial asymmetries from posteroanterior cephalograms: validity of reference lines. Am J Orthod Dentofacial Orthop.

[3] Yoon Y-J, Kim D-H, Yu P-S, Kim H-J, Choi E-H, Kim K-W (2002). Effect of head rotation on posteroanterior cephalometric radiographs. Angle Orthod.

[4] Grummons DC, Kappeyne van de Coppello MA (1987). A frontal asymmetry analysis. J Clin Orthod.

[5] Kim YH, Sato K, Mitani H, Shimizu Y, Kikuchi M (2003). Asymmetry of the sphenoid bone and its suitability as a reference for analyzing craniofacial asymmetry. Am J Orthod Dentofacial Orthop.

[6] Letzer GM, Kronman JH (1967). A posteroanterior cephalometric evaluation of craniofacial asymmetry. Angle Orthod.

[7] Lee J-K, Jung P-K, Moon C-H (2014). Three-dimensional cone beam computed tomographic image reorientation using soft tissues as reference for facial asymmetry diagnosis. Angle Orthod.

[8] Hwang H, Yuan D, Jeong K, Uhm G, Cho J, Yoon S (2012). Three-dimensional soft tissue analysis for the evaluation of facial asymmetry in normal occlusion individuals. Korean J Orthod.

[9] Kook Y-A, Kim Y (2011). Evaluation of facial asymmetry with three-dimensional cone-beam computed tomography. J Clin Orthod.

[10] Meyer-Marcotty P, Stellzig-Eisenhauer A, Bareis U, Hartmann J, Kochel J (2011). Three-dimensional perception of facial asymmetry. Eur J Orthod.

[11] Hwang H-S, Hwang CH, Lee K-H, Kang B-C (2006). Maxillofacial 3-dimensional image analysis for the diagnosis of facial asymmetry. Am J Orthod Dentofacial Orthop.

[12] You Kug-Ho, Lee Kee-Joon, Lee Sang-Hwy, Baik Hyoung-Seon (2010). Three-dimensional computed tomography analysis of mandibular morphology in patients with facial asymmetry and mandibular prognathism. American Journal of Orthodontics and Dentofacial Orthopedics.

[13] Katsumata A, Fujishita M, Maeda M, Ariji Y, Ariji E, Langlais RP (2005). 3D-CT evaluation of facial asymmetry. Oral Surg Oral Med Oral Pathol Oral Radiol Endod.

[14] Gateno J, Xia JJ, Teichgraeber JF (2011). New 3-dimensional cephalometric analysis for orthognathic surgery. J Oral Maxillofac Surg.

[15] Netherway DJ, Abbott AH, Gulamhuseinwala N, McGlaughlin KL, Anderson PJ, Townsend GC, David DJ (2006). Three-dimensional computed tomography cephalometry of plagiocephaly: asymmetry and shape analysis. Cleft Palate Craniofac J.

[16] Maeda M, Katsumata A, Ariji Y, Muramatsu A, Yoshida K, Goto S, Kurita K, Ariji E (2006). 3D-CT evaluation of facial asymmetry in patients with maxillofacial deformities. Oral Surg. Oral Med Oral Pathol Oral Radiol Endod.

[17] Yáñez-Vico RM, Iglesias-Linares A, Torres-Lagares D, Gutiérrez-Pérez JL, Solano-Reina E (2011). Three-dimensional evaluation of craniofacial asymmetry: an analysis using computed tomography. Clin Oral Investig.

[18] Yoon S-J, Lim H-J, Byung-Cheol Kang H-SH (2007). Three dimensional CT analysis of facial asymmetry. Korean J Oral Maxillofac Radiol.

[19] Adams GL, Gansky SA, Miller AJ, Harrell WE, Hatcher DC (2004). Comparison between traditional 2-dimensional cephalometry and a 3-dimensional approach on human dry skulls. Am J Orthod Dentofacial Orthop.

[20] Choi J-H, Mah J (2010). A new method for superimposition of CBCT volumes. J Clin Orthod.

[21] Masuoka N, Muramatsu A, Ariji Y, Nawa H, Goto S, Ariji E (2007). Discriminative thresholds of cephalometric indexes in the subjective evaluation of facial asymmetry. Am J Orthod Dentofacial Orthop.

[22] Manara R, Schifano G, Brotto D, Mardari R, Ghiselli S, Gerunda A, Ghirotto C, Fusetti S, Piacentile K, Scienza R, Ermani M, Martini A (2016). Facial asymmetry quantitative evaluation in oculoauriculovertebral spectrum. Clin Oral Investig.

[23] Haraguchi S, Takada K, Yasuda Y (2002). Facial asymmetry in subjects with skeletal Class III deformity. Angle Orthod.

[24] Ghang M-H, Kim H-M, You J-Y, Kim B-H, Choi J-P, Kim S-H, Choung P-H (2013). Three-dimensional mandibular change after sagittal split ramus osteotomy with a semirigid sliding plate system for fixation of a mandibular setback surgery. Oral Surg Oral Med Oral Pathol Oral Radiol.

[25] Houston WJ (1983). The analysis of errors in orthodontic measurements. Am J Orthod.

[26] Damstra J, Oosterkamp BCM, Jansma J, Ren Y (2011). Combined 3-dimensional and mirror-image analysis for the diagnosis of asymmetry. Am J Orthod Dentofacial Orthop.

[27] Ackerman JL, Proffit WR, Sarver DM, Ackerman MB, Kean MR (2007). Pitch, roll, and yaw: describing the spatial orientation of dentofacial traits. Am J Orthod Dentofacial Orthop.

[28] Park JU, Kook Y-A, Kim Y (2012). Assessment of asymmetry in a normal occlusion sample and asymmetric patients with three-dimensional cone beam computed tomography: a study for a transverse reference plane. Angle Orthod.

[29] Kwon SM, Hwang JJ, Jung Y-H, Cho B-H, Lee K-J, Hwang C-J, Choi S-H (2019). Similarity index for intuitive assessment of three-dimensional facial asymmetry. Sci Rep.

[30] Chien PC, Parks ET, Eraso F, Hartsfield JK, Roberts WE, Ofner S (2009). Comparison of reliability in anatomical landmark identification using two-dimensional digital cephalometrics and three-dimensional cone beam computed tomography in vivo. Dentomaxillofac Radiol.

